# Anomalous isotope effect in iron-based superconductors

**DOI:** 10.1038/s41598-019-42041-z

**Published:** 2019-04-03

**Authors:** Wen-Min Huang, Hsiu-Hau Lin

**Affiliations:** 1 Department of Physics, National Chung Hsing University, 40227 Taichung, Taiwan; 2 Department of Physics, National Tsing Hua University, 30013 Hsinchu, Taiwan

## Abstract

The role of electron-phonon interactions in iron-based superconductor is currently under debate with conflicting experimental reports on the isotope effect. To address this important issue, we employ the renormalization-group method to investigate the competition between electron-electron and electron-phonon interactions in these materials. The renormalization-group analysis shows that the ground state is a phonon-dressed unconventional superconductor: the dominant electronic interactions account for pairing mechanism while electron-phonon interactions are subdominant. Because of the phonon dressing, the isotope effect of the critical temperature can be normal or reversed, depending on whether the retarded intra- or inter-band interactions are altered upon isotope substitutions. The connection between the anomalous isotope effect and the unconventional pairing symmetry is discussed at the end.

## Introduction

Superconductivity^1–5^ is a novel phenomenon of zero electric resistance in some materials when cooled below the characteristic critical temperature *T*_*c*_. The magic arises from electron pairing in superconductors such that the low-energy excitations are described by an exotic quantum condensate without any dissipation. In conventional superconductors, such as aluminium, the interactions between electrons and the lattice vibrations generate effective attraction and lead to electron pair formation. In quantum language, these vibrations can be treated as particle-like excitations named phonons. It is generally believed that the electron-phonon interactions explain the pairing mechanism for conventional superconductors.

On the contrary, the pairing mechanism of the unconventional superconductors, such as cuprates, seems to stem from the strong electron-electron interactions. Despite of intensive experimental and theoretical studies^1,2^ in the past decades, there are still plenty of unsettled controversies about these unconventional superconductors. One of the most important issues is the interplay between the electron-electron and the electron-phonon interactions^6–18^. The recently discovered iron-based superconductors^3–5,19–23^ provide a unique testing ground to address this issue^24–27^. Gathered from theoretical and experimental investigations, the interaction strength in the iron-based superconductors is only weak to medium, rendering controlled theoretical understanding possible.

One of the checking points is the critical temperature of superconductivity upon isotope substitutions^28–31^. According to the Bardeen-Cooper-Schrieffer theory for the conventional superconductors, the critical temperature *T*_*c*_ is related to the mass of the isotope element *M*,

1 $${T}_{c}\sim {M}^{-\alpha },$$

where *α* is the exponent for the isotope effect. If the dominant interaction is electron-phonon in nature, theoretical calculations give $\alpha =1/2$. In the extreme opposite, if the pairing is completely driven by electron-electron interactions, the critical temperature should not change with isotope substitutions and the corresponding exponent is $\alpha \approx 0$. In realistic superconductors, we expect the isotope exponent to be in-between. Note that, in unconventional superconductors, the phonon-mediated interactions are insufficient to explain the pairing mechanism and it is of crucial importance to study the interplay between electron-electron and electron-phonon interactions^24–27^. For instance, even when the pairing mechanism is electronic origin, dispersions observed in angle-resolved photoemission spectroscopy manifest distortions upon isotope substitutions^13–18,27^.

The isotope effect observed in iron-based superconductor^28–34^ seems to tell a more complicated story. For instance, a strong isotope effect by iron substitution^28^ is found in SmFeAs(O, F) and (Ba, K)Fe_2_As_2_, almost as large as that in conventional superconductors. On the contrary, *inverse* isotope effect^29^ is spotted in (Ba, K)Fe_2_As_2_ with different isotope substitutions. Later, it was proposed that the isotope substitutions may give rise to structural change^35^ and further complicate the story. On the theoretical side, Yanagisawa *et al*.^36^ proposed a multi-band and multi-channel model to explain the possibility of observing the inverse isotope effect. However, Bussmann-Holder and Keller^37^ commented that an inversion of the exponent cannot occur upon iron isotope substitutions. The controversies about the isotope effect of the iron-based superconductor are still on. And, it is of crucial importance to clarify the subtle role of the electron-phonon interactions in iron-based superconductors.

## Results

### Instantaneous and retarded interactions

**Figure 1:**
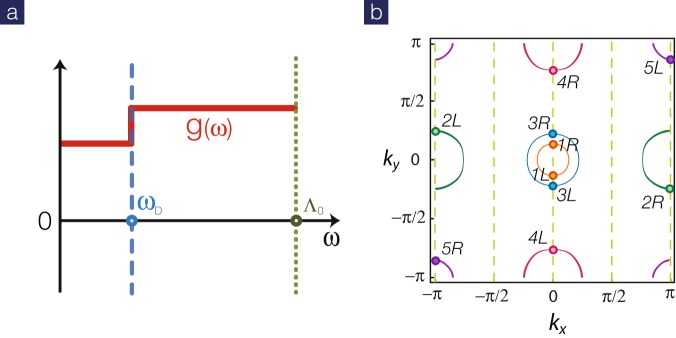
(**a**) Step-like interaction profile for simultaneous and retarded interactions. A sharp step is assumed at the Debye frequency ${\omega }_{D}$. (**b**) Fermiology of the five-band model $x=0.1$. These Fermi surfaces are well sampled by five pairs of Fermi points, equivalent to the four-leg geometry with quantized momenta (dashed lines).

Motivated by the controversy, we investigate the competition between electron-electron and electron-phonon interactions by the unbiased renormalization-group (RG) method. Due to the retarded nature of the phonon-mediated interactions, the energy dependence must be included. The minimal approach to include both simultaneous and retarded interactions can be accomplished by the step-shape approximation^38–41^ as shown in Fig. 1(a),

2 $${g}_{i}(\omega )={g}_{i}+{\tilde{g}}_{i}{\rm{\Theta }}({\omega }_{D}-\omega ),$$

where *g*_*i*_ and ${\tilde{g}}_{i}$ represent (instantaneous) electronic interactions and (retarded) phonon-mediated ones. The energy scale for the retarded interactions is set by the Debye frequency ${\omega }_{D}$. Our RG analysis reveals that the pairing mechanism is dominated by the electronic interactions *g*_*i*_. But, the retarded interactions ${\tilde{g}}_{i}$ also grow under RG transformation and become relevant in low-energy limit. Inclusion of these subdominant interactions leads to anomalous isotope effect. The isotope exponent *α* can be extracted numerically from RG flows in weak coupling. It is quite remarkable that the sign of the exponent *α* sensitively depends on whether the inter- and/or intra-band interactions are altered by isotope substitutions.

### Multi-band model

To illustrate how the RG scheme works, we start with a five-orbital tight-binding model for iron-based superconductors with generalized on-site interactions,

3 $$\begin{array}{rcl}H & = & \sum _{{\boldsymbol{p}},a,b}\,\sum _{\alpha }\,{c}_{{\boldsymbol{p}}a\alpha }^{\dagger }{K}_{ab}({\boldsymbol{p}}){c}_{{\boldsymbol{p}}b\alpha }+{U}_{1}\,\sum _{i,a}\,{n}_{ia\uparrow }{n}_{ia\downarrow }\\  &  & +\,{U}_{2}\,\sum _{i,a < b}\,\sum _{\alpha ,\beta }\,{n}_{ia\alpha }{n}_{ib\beta }+{J}_{H}\,\sum _{i,a < b}\,\sum _{\alpha ,\beta }\,{c}_{ia\alpha }^{\dagger }\,{c}_{ib\alpha }\,{c}_{ib\beta }^{\dagger }\,{c}_{ia\beta }\\  &  & +\,{J}_{H}\,\sum _{i,a < b}\,[{c}_{ia\uparrow }^{\dagger }{c}_{ia\downarrow }^{\dagger }{c}_{ib\downarrow }{c}_{ib\uparrow }+{\rm{H}}.\,{\rm{c}}.\,]\},\end{array}$$

where $a,b=1,2,\ldots ,5$ label the five *d*-orbitals of Fe, $1:{d}_{3{Z}^{2}-{R}^{2}}$, $2:{d}_{XZ}$, $3:{d}_{YZ}$, $4:{d}_{{X}^{2}-{Y}^{2}}$, $5:{d}_{XY}$, and *α* = ↑,↓ is the spin index. The kinetic matrix *K*_*ab*_ in the momentum space has been constructed in previous studies^42^. The generalized on-site interactions consist of three parts: intra-orbital *U*_1_, inter-orbital *U*_2_ and Hund’s coupling *J*_*H*_. Adopted from previous studies, we choose the values, ${U}_{1}=4\,{\rm{eV}}$, ${U}_{2}=2\,{\rm{eV}}$ and ${J}_{H}=0.7\,{\rm{eV}}$ for numerical studies here.

Fermiology is important in the multi-band superconductors. The electron doping *x* is related to the band filling $n=6+x$ ($n=10$ for completely filled bands) here and the Fermi surface at $x=0.1$ is illustrated in Fig. 1(b). There are five active bands: two hole pockets centered at (0, 0) and another hole pocket centered at (*π*, *π*) while two electron pockets located at (*π*, 0)and (0, *π*) points^43^. To simplify the RG analysis, we sample each pocket with one pair of Fermi points (required by time-reversal symmetry). This is equivalent to a four-leg ladder geometry with quantized momenta as shown in Fig. 1(b). In the low-energy limit, the effective Hamiltonian^44–46^ is captured by five pairs of chiral fermions with different velocities. The RG equations for all couplings can be found in Methods.

### Pairing mechanism

**Figure 2:**
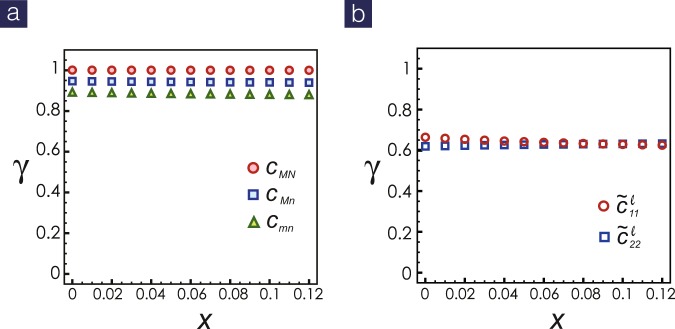
RG exponents for (**a**) the simultaneous and (**b**) the retarded Cooper scatterings. The dominant interactions are pairing hopping between and within band 1 and band 2, with maximal exponent of unity, while other relevant couplings are subdominant with RG exponent smaller than one.

By integrating the two sets of RG equations numerically, we found all couplings are well described the scaling ansatz^47^,

4 $${g}_{i}\approx \frac{{G}_{i}}{{({l}_{d}-l)}^{{\gamma }_{{g}_{i}}}},\,{\tilde{g}}_{i}\approx \frac{{\tilde{G}}_{i}}{{({l}_{d}-l)}^{{\gamma }_{{\tilde{g}}_{i}}}},$$

where *G*_*i*_, ${\tilde{G}}_{i}$ are non-universal constants and ${\gamma }_{{g}_{i}}$, ${\gamma }_{{\tilde{g}}_{i}}$ are RG exponents for simultaneous and retarded couplings. The divergent length scale *l*_*d*_, associated with the pairing gap, is solely determined by electronic origin. The dominant pairing occur within band 1 and band 2 and the Cooper scatterings *c*_11_, *c*_22_ *c*_12_ have maximum exponent ${\gamma }_{i}=1$. Other Cooper scatterings are subdominant with exponents close to 0.9, as shown in Fig. 2(a). Meanwhile, by Abelian bosonization^45,46^, the signs of *c*_*ij*_ from numerics lead to sign-revised (between electron and hole pockets) *s*_±_-wave pairing, agreeing with the previous functional RG study^48^. Note that these exponents are rather robust within the doping range where the same Fermiology maintains. What about the phonon-mediated interactions? As clearly indicated in Fig. 2(b), the RG exponents for ${\tilde{c}}_{11}$, ${\tilde{c}}_{22}$ are roughly 0.6, much smaller than the dominant electronic interactions, showing the pairing mechanism is electronic origin. However, since the RG exponents are positive, the retarded interactions also grow under RG transformation. These subdominant phonon-mediated interactions can lead to anomalous isotope effect as explained in the following.

### Two-step RG scheme

**Figure 3:**
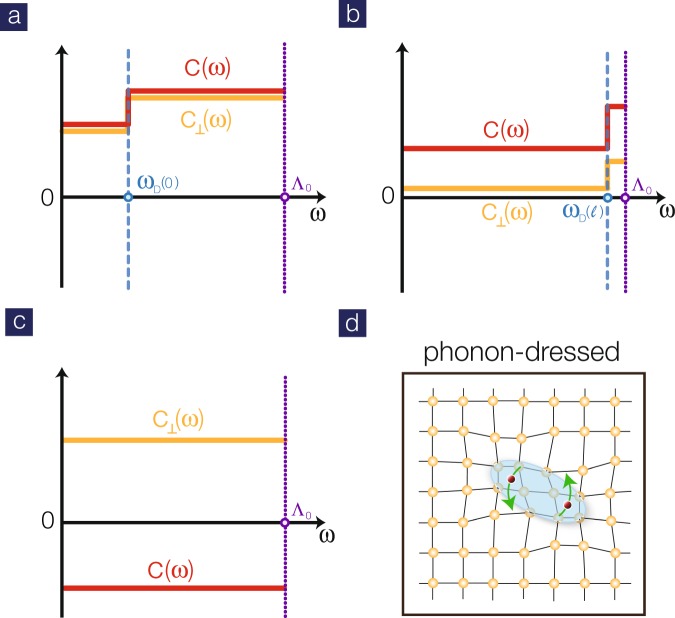
(**a**) Interaction profile of the dominant intra-band *C* and inter-band *C*_⊥_ Cooper scatterings before RG transformation. (**b**) As RG progresses, the step evolves since the Debye energy is rescaled, ${\omega }_{D}(l)={\omega }_{D}{e}^{l}$. (**c**) For $l > {l}_{D}$, the distinction between simultaneous and retarded interactions disappears. (**d**) Schematic picture for phonon-dressed unconventional superconductor.

To achieve quantitative understanding in weak coupling, the rescaled Debye frequency must be taken into account carefully. Under RG transformations, ${\omega }_{D}\to {\omega }_{D}{e}^{l}$ as shown in Fig. 3. At the (logarithmic) length scale ${l}_{D}\equiv \,\mathrm{log}({{\rm{\Lambda }}}_{0}/{\omega }_{D})$, the difference between *g*_*i*_ and ${\tilde{g}}_{i}$ disappears. The Debye frequency ${\omega }_{D}\sim 30\,{\rm{meV}}$ in iron-based materials^24^ and the band width (thus Λ_0_) is 3–4 eV, giving rise to *l*_*D*_ ~ 5. Note that the RG is truncated at the cutoff length scale *l*_*c*_ where the maximal coupling reaches order one. In weak coupling, it is clear that ${l}_{c} > {l}_{D}$ and thus the RG scheme must be divided into two steps. For $l < {l}_{D}$, both sets of RG equations are employed. At $l={l}_{D}$, the functional form for the retarded interactions is the same as the instantaneous one. Thus, one should add up both types of couplings ${g}_{i}({l}_{D})+{\tilde{g}}_{i}({l}_{D})$ and keep running RG by just the first set of equations. In physics terms, this means that the difference between simultaneous and retarded interactions vanishes before the pairing gaps open.

### Extracting isotope exponent

Numerical results for the two-step RG indicate the same superconducting phase as described in previous paragraphs but the isotope exponent *α* can be extracted numerically. Under RG transformation, the critical temperature satisfies the scaling form, ${k}_{B}{T}_{c}\sim {\rm{\Delta }}\,[g(0)]={{\rm{\Delta }}}_{c}{e}^{-{l}_{c}}$, where Δ_*c*_ is the pairing gap at the cutoff length scale. By varying the length scale *l*_*D*_, the critical temperature changes, i.e.

5 $$\frac{d(\mathrm{log}\,{T}_{c})}{d{l}_{D}}\approx \frac{d(\mathrm{log}\,{{\rm{\Delta }}}_{c})}{d{l}_{D}}-\frac{d{l}_{c}}{d{l}_{D}}.$$

Furthermore, from the definition of the isotope exponent, the standard scaling argument under RG transformation gives rise to the isotope exponent.

6 $$\alpha \equiv -\,\frac{d(\mathrm{log}\,{T}_{c})}{d(\mathrm{log}\,M)}\approx \frac{1}{2}\frac{d(\mathrm{log}\,{{\rm{\Delta }}}_{c})}{d(\mathrm{log}\,{\omega }_{D})}+\frac{1}{2}\frac{d{l}_{c}}{d{l}_{D}},$$

where $d(\mathrm{log}\,M)=-\,2d(\mathrm{log}\,{\omega }_{D})=2d{l}_{D}$, because ${\omega }_{D}\sim {M}^{-1/2}$. The above formula for the isotope exponent *α* is the central result in this paper. For conventional superconductor, ${{\rm{\Delta }}}_{c}\sim {\omega }_{D}$ and the cutoff length scale is not sensitive to the Debye frequency (the second term vanishes). Thus, $\alpha \approx 1/2$. On the other hand, for unconventional superconductors without relevant electron-phonon interactions, ${{\rm{\Delta }}}_{c}\sim {{\rm{\Lambda }}}_{0}$ and the cutoff length scale is also not sensitive to the Debye frequency. It is clear that *α* = 0 in this case. But, what happens if the electron-phonon interactions, though not dominant, are actually relevant under RG transformation? We shall elaborate the details in Discussion.

## Discussion

**Figure 4:**
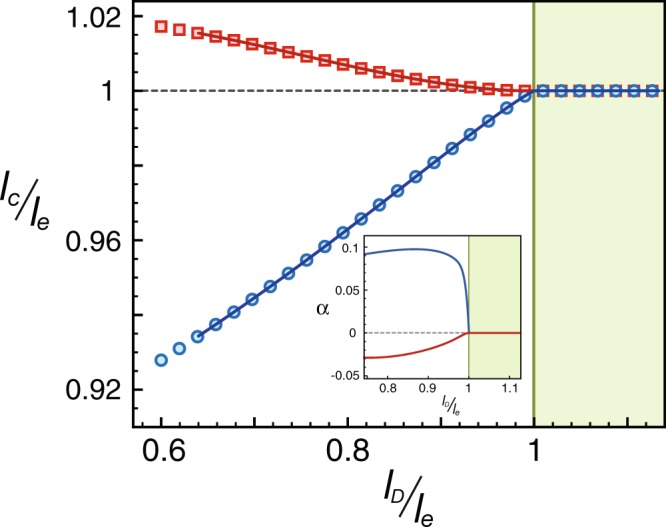
The cutoff length scale *l*_*c*_ versus *l*_*D*_ for inclusion of intraband interactions ${\tilde{c}}_{ii}(0)=-\,0.3\,U$ (blue circles) and interband ones ${\tilde{c}}_{ij}(0)=-\,0.14\,U$ (red square), where *U* is the strength of electron-electron interactions. For convenience, the axes are rescaled in the unit of *l*_*e*_, the cutoff length scale with electronic interactions only. The inset shows the isotope exponent by taking numerical derivative.

To extract the isotope exponent, we study how the cutoff length scale *l*_*c*_ varies with different Debye frequencies due to isotope substitutions. In weak coupling, we found that *g*_*i*_ are much larger than ${\tilde{g}}_{i}$. Thus, Δ_*c*_ has very weak dependence on ${\omega }_{D}$ and the first term can be ignored. The contribution from the second term is shown in Fig. 4. We tried two different profiles for the retarded interactions. Include only intra-band interactions, ${\tilde{c}}_{ii}(0)=-\,0.3\,U$ first, where *U* is the strength of electron-electron interactions. The isotope exponent is positive (reading from the slope), $\alpha \approx 0.1$, with very smooth variation. On the other hand, with only inter-band interactions, ${\tilde{c}}_{ij}(0)=-\,0.14\,U$, the isotope exponent is negative and changes gradually from zero to $\alpha \approx -\,0.03$.

These anomalous isotope effects are closely related to the unconventional pairing symmetry. For the *s*_**±**_-wave pairing, ${c}_{ii} < 0$ but ${c}_{ij} > 0$ at the cutoff length scale. The phonon-mediated intra-band interactions ${\tilde{c}}_{ii} < 0$ help to develop the pairing instability and thus lead to a positive isotope exponent. On the other hand, the inter-band ones ${\tilde{c}}_{ij} < 0$ have opposite sign with their simultaneous counterparts *c*_*ij*_. In consequence, the pairing instability is suppressed and an inverses isotope effect is in order. The RG analysis presented here provides clear and natural connection between the anomalous isotope effect and the unconventional pairing symmetry.

Although the isotope exponent *α* can be extracted numerically in weak coupling, extending the quantitative description to intermediate coupling may not be easy. If the pairing gaps open before hitting the Debye energy scale, i.e. ${l}_{c} < {l}_{D}$, our numerical results show that *l*_*c*_ solely depend on electronic interactions and thus $d{l}_{c}/d{l}_{D}=0$. The isotope exponent in this regime mainly arises from the first term. The pairing gap ${{\rm{\Delta }}}_{c}={{\rm{\Delta }}}_{c}({{\rm{\Lambda }}}_{0},{\omega }_{D}{e}^{l})$, depending on both the bandwidth and the rescaled Debye frequency, is now quite complicated. The RG analysis alone is not sufficient to obtain *α* in a quantitative fashion. However, we recently found that the effective Hamiltonian at the cutoff length scale is well captured by mean-field theory (not yet published). In principle, one can combine RG and mean-field approaches together to compute the isotope exponent in intermediate coupling more accurately.

In the end, we discuss the recent discovery of superconductivity in FeSe/STO systems^49,50^. We emphasize that our current approach includes fermiology, electron-electron interactions, and electron-phonon interactions within only the superconducting(SC) layers. One crucial assumption is the profile of the mediated electron-phonon interactions can be captured by the step function. The RG scheme built upon this approximation works as explained in the manuscript. However, according to the recent literatures in FeSe/STO systems^49–56^, to include the non-SC (SrTiO_3_) layers we need to devise a new theoretical approach which is beyond our model at this point. The profile of the electron-phonon interactions arisen from non-SC layers is probably not captured by the simple step function anymore. One needs to find out the interaction profile generated by the non-SC layers first so that one can devise the RG scheme accordingly. This is going to be an interesting and challenging topic to explore in the future.

## Methods

### RG equations

The interactions between these chiral fermions fall into two categories^39^: Cooper scattering ${c}_{ij}^{l}$, ${c}_{ij}^{s}$ and forward scattering ${f}_{ij}^{l}$, ${f}_{ij}^{s}$. The retarded ones share the same classification, denoted with an extra tilde symbol. The RG equations for the simultaneous interactions are,

7 $$\begin{array}{rcl}{\dot{c}}_{ii}^{l} & = & -2\,\sum _{k\ne i}\,{\alpha }_{ii,k}{c}_{ik}^{l}{c}_{ki}^{s}-2\,{({c}_{ii}^{l})}^{2},\\ {\dot{c}}_{ii}^{s} & = & -\sum _{k\ne i}\,{\alpha }_{ii,k}\,[{({c}_{ik}^{l})}^{2}+{({c}_{ik}^{s})}^{2}]-{({c}_{ii}^{l})}^{2},\\ {\dot{c}}_{ij}^{l} & = & -\sum _{k}\,{\alpha }_{ij,k}\,[{c}_{ik}^{l}{c}_{kj}^{s}+{c}_{jk}^{l}{c}_{ki}^{s}]-4{f}_{ij}^{l}{c}_{ij}^{l}\\  &  & +\,2{f}_{ij}^{l}{c}_{ij}^{s}+2{f}_{ij}^{s}{c}_{ij}^{l},\\ {\dot{c}}_{ij}^{s} & = & -\sum _{k}\,{\alpha }_{ij,k}\,[{c}_{ik}^{l}{c}_{kj}^{l}+{c}_{ik}^{s}{c}_{kj}^{s}]+2{f}_{ij}^{s}{c}_{ij}^{s},\\ {\dot{f}}_{ij}^{l} & = & -2\,{({f}_{ij}^{l})}^{2}-2\,{({c}_{ij}^{l})}^{2}+2{c}_{ij}^{l}{c}_{ij}^{s},\\ {\dot{f}}_{ij}^{s} & = & {({c}_{ij}^{s})}^{2}-{({f}_{ij}^{l})}^{2},\end{array}$$

where $\dot{g}=dg/dl$, where $l=\,\mathrm{ln}({{\rm{\Lambda }}}_{0}/{\rm{\Lambda }})$ is the logarithm of the ratio between bare energy cutoff ${{\rm{\Lambda }}}_{0}$ and the running cutoff ${\rm{\Lambda }}$. The tensor ${\alpha }_{ij,k}=({v}_{i}+{v}_{k})\,({v}_{j}+{v}_{k})/[2{v}_{k}({v}_{i}+{v}_{j})]$ with *v*_*i*_ representing the Fermi velocities.

The second set of equations describes how the retarded interactions are renormalized,

8 $$\begin{array}{rcl}{\dot{\tilde{c}}}_{ii}^{l} & = & 2{\tilde{c}}_{ii}^{l}{c}_{ii}^{s}-4{\tilde{c}}_{ii}^{l}{c}_{ii}^{l}-2\,{({\tilde{c}}_{ii}^{l})}^{2},\\ {\dot{\tilde{c}}}_{ij}^{l} & = & -4{\tilde{f}}_{ij}^{l}{c}_{ij}^{l}-4{f}_{ij}^{l}{\tilde{c}}_{ij}^{l}-4{\tilde{f}}_{ij}^{l}{\tilde{c}}_{ij}^{l}+2{\tilde{f}}_{ij}^{l}{c}_{ij}^{s}+2{f}_{ij}^{s}{\tilde{c}}_{ij}^{l},\\ {\dot{\tilde{f}}}_{ij}^{l} & = & -4{\tilde{f}}_{ij}^{l}{f}_{ij}^{l}-2\,{({\tilde{f}}_{ij}^{l})}^{2}-4{\tilde{c}}_{ij}^{l}{c}_{ij}^{l}-2\,{({\tilde{c}}_{ij}^{l})}^{2}\\  &  & +\,2{\tilde{f}}_{ij}^{l}{f}_{ij}^{s}+{\tilde{c}}_{ij}^{l}{c}_{ij}^{s}.\end{array}$$

Note that we separate the intra-band and inter-band couplings for clarity, i.e. $i\ne j$ in the above RG equations. In fact, the separation is necessary because we shall see later that inter-band and intra-band couplings play different roles in the low-energy limit. In addition, ${f}_{ii}=0$ and ${\tilde{f}}_{ii}=0$ to avoid double counting.
